# Dynamics of Preparatory Apneas and Their Influence on Maximal Dry Static Apnea in Breath-Hold Divers

**DOI:** 10.3390/jfmk10040471

**Published:** 2025-12-04

**Authors:** Dario Vrdoljak, Colin D. Hubbard, Geoff B. Coombs, Andrew T. Lovering, Ivan Drvis, Nikola Foretic, Joseph W. Duke, Željko Dujić

**Affiliations:** 1 Centre for Heart, Lung and Vascular Health, University of British Columbia, Okanagan Campus, Kelowna, BC V1V 1V7, Canada; dariov.vrdoljak@ubc.ca; 2 Department of Biological Sciences, Northern Arizona University, Flagstaff, AZ 86011, USA; cdh294@nau.edu (C.D.H.); jj.duke@nau.edu (J.W.D.); 3 School of Psychology and Sport Science, Bangor University, Bangor LL57 2DG, UK; g.coombs@bangor.ac.uk; 4 Department of Human Physiology, University of Oregon, Eugene, OR 97403, USA; lovering@uoregon.edu; 5 Faculty of Kinesiology, University of Zagreb, 10000 Zagreb, Croatia; ivan.drvis@kif.unizg.hr; 6 Faculty of Kinesiology, University of Split, 21000 Split, Croatia; nikola.foretic@kifst.eu; 7 Department of Integrative Physiology, University of Split School of Medicine, 21000 Split, Croatia

**Keywords:** preconditioning, breath-holding, diaphragm pressure, involuntary breathing movements, electromyography

## Abstract

**Background:** Physiological and psychological factors are important for determining static breath-hold duration. Preconditioning, such as preparatory apneas at functional residual capacity, is a potentially valuable method for prolonging breath-hold duration at total lung capacity. We investigated the physiological influence of preparatory apneas to determine the possible association with maximal apnea duration via diaphragmatic pressure and electromyographic measurements. **Methods**: Fourteen male breath-hold divers (39 ± 10 years; body mass, 87.2 ± 8.5 kg; body fat, 14.4 ± 3.8%; body height, 186.6 ± 3.9 cm; training experience, 14.2 ± 9.6 years) were included. We measured diaphragm activity during breath-holds via transdiaphragmatic pressure (Pdia) using balloon-tipped catheters in the stomach and esophagus and electromyographic (EMG) activity. From these, ∆EMG and ∆Pdia for every involuntary breathing movement (IBM) during all apneas were quantified. Furthermore, a pressure difference (difference between the first and last IBM Pdia value) and the respiratory neuromuscular output index (RNMI) (∆Pdia/∆EMG) were included as indirect parameters of the pressure perceived. These variables were measured during three preparatory breath-holds (average duration = 185 ± 69 s and range = 62–309 s, separated by 2.5 min) and three maximal breath-holds (average duration = 308 s and range = 179–733 s, separated by 5 min). **Results**: The preparatory apnea performed at FRC elicited significantly higher Pdia activity (*p* < 0.00) and a significantly lower RNMI (*p* = 0.00–0.01) compared to the maximal apneas. Furthermore, a higher and more pronounced increase in Pdia during the preparatory apnea at FRC was related to longer maximal apneas (Max 1, r = 0.65, *p* = 0.01; Max 2, r = 0.65, *p* = 0.02; Max 3, r = 0.66, *p* = 0.01). **Conclusions**: The results suggest an acute preconditioning effect of primarily the preparatory apnea at FRC on the duration of the subsequent maximal apneas. The implementation of preparatory apneas preceding maximal apneas during training sessions may elicit a longer breath-hold duration in trained divers.

## 1. Introduction

Breath-hold diving (BHD) is an activity in which the participants endure a hypoxic environment for a prolonged time. At the elite level, freedivers can hold their breath for an impressive duration of ~10 min and can reach depths of over 200 m, depending on the diving discipline [1]. Previous work has reviewed the components of a maximal, static apnea in elite divers Bain et al. [2]. This prior work noted that the maximal static breath-hold duration is primarily determined by the following factors: preparation (warm-up apneas, breathing techniques), maximal oxygen storage, minimal oxygen consumption, minimal CO_2_ accumulation, and motivation [2]. These factors are increased and improved with both apneic training and preconditioning (preparation) preceding a maximal breath-hold.

One major physiological factor that can improve the maximal breath-hold duration is increasing lung volume and/or the volume of O_2_ in the lungs at the start of the breath-hold. One way of achieving this is through performing glossopharyngeal insufflation [2,3]. Additionally, during prolonged breath holds, divers experience the mammalian diving response (DR) [4,5], which includes cardiovascular adjustments that lead to a reduction in oxygen consumption by peripheral tissues, thereby ensuring a sufficient oxygen supply for the vital organs (brain and heart) [6]. It has been shown that trained apneists have a larger DR compared with untrained control subjects, as shown by a greater decrease in heart rate (HR) and a larger increase in mean arterial pressure (MAP), both of which influence breath-hold duration [7].

Moreover, increased motivation, improved relaxation techniques and stress tolerance through training can influence breath-hold duration [8]. These are necessary due to the events during a maximal voluntary apnea. A maximal apnea has two phases: the initial or easy-going phase and the struggle phase. The easy-going phase is “easy” and ends when CO_2_ accumulation reaches a threshold (PaCO_2_ threshold level of ~6.5 ± 0.5 kPa [9]). During the easy-going phase, there is an increasing urge to breathe, and voluntary suppression of respiratory neuromuscular output can no longer be maintained. This is when involuntary breathing movements (IBMs) occur and mark the onset of the struggle phase. During IBMs, there is contraction of both inspiratory and expiratory muscles [6]. Additionally, during IBMs, stroke volume is augmented and cardiac output is normalized via increased inferior vena cava blood flow [10]. For more details on the respiratory responses to a maximal breath-hold, please see the recent review by Hubbard et al. [11].

One thing that may improve the duration of dry, static, maximal breath-holds is preconditioning that includes preparatory apneas [12]. However, there is no empirical evidence that preparatory apneas improve maximal apnea duration. Nevertheless, divers have been performing preparatory apneas as part of our studies for years. The protocol in the current study, developed by a member of the research team who is a former national diving coach (ID), hypothesizes that the preparatory apneas could lead to the ‘softening’ of the diaphragm so that divers could endure the struggle phase with more ease. Thus, preparation of the respiratory neuromuscular output for the upcoming prolonged apneas may be influenced by proper preparation. Also, the preparatory apneas may serve solely as a warm-up technique, which is known to have a positive influence on muscular performance by enhancing the speed of calcium release from the sarcoplasmic reticulum. Therefore, this study aimed to investigate the physiological influence of preparatory apneas and to determine the possible influence on maximal apnea prolongation through diaphragmatic pressure and electromyographic measurements.

## 2. Materials and Methods

### 2.1. Participants

The experimental group consisted of fourteen breath-hold divers. At the time of the study, three participants were national team competitors who participated in international competitions (one is a previous world record holder), and the others are considered moderate BHD athletes. At the time of the study, they were all apparently healthy. Participants completed a pre-study health questionnaire, which determined no previous illnesses or medication use that would exclude them from the study. Anthropometric and lung function data of the participants are shown in Table 1.

### 2.2. Experimental Design

All experimental procedures were performed following the Declaration of Helsinki on the treatment of human subjects and were approved by the Ethical Committee of the University of Split Faculty of Kinesiology (ethics board approval no. 2181-205-02-05-24-007). Informed, written consent was obtained from each subject. All experiments were carried out in a climate-controlled room (22–24 °C), as was performed following previously defined procedures [13]. The participants arrived at the laboratory 30–45 min before the start of the experiments for instrumentation and an explanation of the procedures. They had abstained from food for at least 4 h and had approximately 30 min of quiet rest before the start of the experiment.

**Figure 1:**
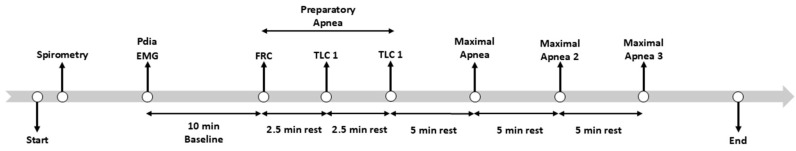
Protocol timeline. FRC, functional residual capacity; TLC, total lung capacity, EMG, electromyography; Pdia, diaphragmatic pressure.

The experimental procedures are outlined in Figure 1. After obtaining consent, anthropometric data were obtained for each participant (Tanita BC 418; Tokyo, Japan). Next, participants were equipped with two balloon-tipped catheters: one in the stomach and one in the esophagus, as described previously [14] and had EMG electrodes placed to estimate right and left diaphragm hemisphere activity. Then, the participants performed 3 preparatory apneas (1st at functional residual capacity (FRC), 2nd and 3rd at total lung capacity (TLC)), with 2.5 min of rest between. All three preparatory apneas were performed until the participant experienced 7–15 IBMs. This was followed by 3 maximal apneas with 5 min of rest between each. For the whole apnea protocol, participants were allowed to perform glossopharyngeal insufflation (“lung packing”) as they wanted. This study was part of a larger set of experiments, the details of which can be found elsewhere [14].

### 2.3. Anthropometric Indices and Peripheral Estimation of Arterial Oxygen Saturation

Anthropometric data included height, mass, and body fat percentage. Body mass and body fat percentage were assessed with a bioimpedance scale (Tanita BC 418 scale; Tokyo, Japan). Height was determined with a Tanita HR-001 anthropometer (Tanita; Tokyo, Japan).

In n = 6 participants, spirometry was performed using two linear pneumotachographs (4813/3813 series; Hans Rudolph, Shawnee, KS, USA) connected to a data acquisition system (PowerLab 16/35; ADInstruments, Colorado Springs, CO, USA) and visualized on LabChart Pro (v8.1.16; ADInstruments, Colorado Springs, CO, USA). These maneuvers were analyzed, post hoc, manually on Microsoft Excel. In n = 8, spirometry was performed using a computerized spirometer (CPFS/D Spirometer; MedGraphics, St. Paul, MN, USA). Spirometry was conducted and reported using societal standards [14] and using the appropriate predictive equations [15].

Peripheral estimates of arterial oxygen saturation (SpO_2_) and heart rate (HR) were continuously measured using a forehead sensor (Nellcor, Oximax N-600 pulse oximeter, Tyco, Mansfield, MA, USA).

### 2.4. EMG Data Acquisition

EMG electrodes were placed over the 7th and 8th intercostals to represent the diaphragm region. To define the proper position of electrodes, Bilateral Phrenic Nerve Stimulation was performed. Electrical stimulation of the phrenic nerves was performed bilaterally using research-grade electric stimulators (DS7R; Digitimer, Fort Lauderdale, FL, USA). Two probes (Comfort Probe RS10; Carefusion, San Diego, CA, USA) were connected to the stimulators and positioned bilaterally along the phrenic nerve behind the sternocleidomastoid. Delivery of this stimulus elicited a contraction of the diaphragm and was confirmed via EMG signal and transdiaphragmatic pressure changes. The EMG positioning was performed following previously defined procedures [13].

**Figure 2:**
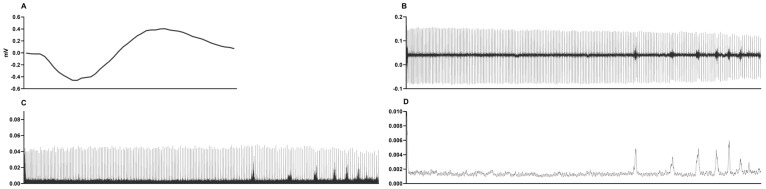
EMG analysis procedure on an individual diver. After electrode placement, Bilateral Phrenic Nerve Stimulation was performed until the M-wave was reached, as presented in (**A**); if the M-wave was not obtained, the electrode placement was adjusted. The raw data (**B**) was analyzed with FFT and a second-order Butterworth bandpass filter (**C**), and normalized (**D**).

The raw EMG data was processed using Fast Fourier Transformation and a second-order Butterworth bandpass filter between 20 and 450 Hz to increase the signal-to-noise ratio. In addition, filtering was performed on the raw data with a root mean square (RMS) filter. The RMS envelope calculation was performed with fully overlapped windows so that the sampling rate of the RMS would be identical to the sampling rate of the acquired data. The normalized signal was averaged in 1000-point windows to obtain clear data (see Figure 2). This process allowed us to detect the IBMs of every apnea individually.

### 2.5. Diaphragmatic Pressure Data Acquisition

Transdiaphragmatic pressure was measured with two balloon-tipped catheters (47-9005; Ackrad Laboratory, Cranford, NJ, USA), one in the stomach (Pga) and one in the esophagus (Poes). After applying a topical anesthetic (2% lidocaine HCl) to numb the naris and nasopharynx, the balloon-tipped catheters were passed, one at a time, through the nares and into the stomach (i.e., ~60–65 cm distal to the nostril tip). Next, the residual air was evacuated from the balloon via a Valsalva maneuver or a forceful cough with an open glottis, after which the balloon was inflated with ~1 to 1.5 mL (esophageal catheter) or 2 to 2.5 mL (gastric catheter) of air [16,17]. After confirming the catheter was positioned in the stomach (i.e., diaphragmatic “bumps” during inspiration), the esophageal catheter was withdrawn until a persistent negative deflection was observed during an inspiratory effort. Final adjustments of catheter position in the esophagus were guided by the “occlusion” test, wherein catheter depth was modified such that the slope of ∆Poes and ∆ mouth pressure (Pmouth) was within 0.98–1.02. Pmouth, Poes, and Pga were measured via differential pressure transducers (range: ±352 cmH_2_O; HSCSNDN005PDAA5, Honeywell International Inc., Morristown, NJ, USA). Pdia was calculated as Pga–Poes. The pressure transducers were calibrated using a digital manometer before the visit.

#### Pdia and RNMI and Pressure Difference Calculation

To determine the relation between nervous system activity and diaphragm pressure, the respiratory neuromuscular output index (RNMI) was calculated using the following equation:

$$RNMI=\frac{P_{dia}}{EMG}$$

where EMG is the electromyography signal amplitude during IBMs and Pdia is the active diaphragm pressure. The RNMI demonstrates the ratio between the central nervous system response and the resulting pressure of the diaphragm, with higher values implying higher EMG activity for smaller Pdia. The pressure difference is the difference between the first and last IBM Pdia value. This equation follows that in a previous study performed by Jansen et al. [18].

### 2.6. Data Acquisition and Statistical Analysis

Data were acquired at 1000 Hz using PowerLab (PowerLab 16/35; ADInstruments, Colorado Springs, CO, USA). Diaphragm electromyographical data was collected using an Octal Bioamp (FE238; ADInstruments, Colorado Springs, CO, USA). The time points of interest were the periods between the onset and end of the apnea. Throughout the apnea phases (e.g., easy-going and struggle), we calculated ΔEMG and ΔPdia for every IBM (the difference between the easy-going phase mean and each IBM maximum) to obtain amplitudes. Additionally, we averaged the amplitude of three IBM ΔEMG and ΔPdia values at the start, in the middle, and at the end. The same calculation was used for the RNMI. Additionally, the mean values during the start, middle, and at the end of the struggle phase of SpO_2_ and HR were calculated.

All data are presented as means ± standard deviation (SD). Comparisons among preparatory and maximal apneas were made via a one-way ANOVA with Fisher LSD post hoc analysis. In the same manner, the phases of the apnea were tested for possible differences. The *p* values in all analyses were tested with the magnitude-based Cohen’s effect size (ES) statistic with modified qualitative descriptors (trivial ES < 0.2; small ES = 0.21–0.60; moderate ES = 0.61–1.20; large ES = 1.21–1.99; and extremely large ES > 2.0).

Statistica version 13.0 (Dell Inc., Austin, TX, USA) was used for the analyses, and a level of 95% (*p* < 0.05) was applied.

## 3. Results

**Figure 3:**
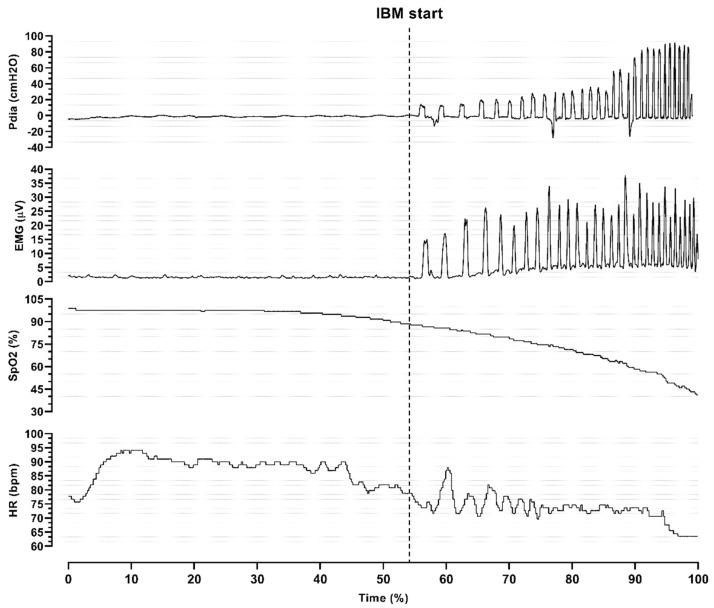
Individual response of various measured parameters during maximal apnea in *diver 1*. Pdia, diaphragmatic pressure; EMG, electromyography signal; SpO_2_, oxygen saturation; HR, heart rate; µV, microvolts; bpm, beats per minute; IBM, involuntary breathing movement.

Analysis of the individual responses during maximal static apnea (Figure 3) revealed that the onset of the struggle phase was followed by higher oxygen desaturation and a decrease in heart rate. Moreover, both Pdia and EMG signals changed, which represented the onset of IBMs.

The struggle phase occupied 37% of the total apnea duration in the preparatory apnea at FRC; 27 and 36% in the two preparatory apneas at TLC; and 38, 34, and 37% of the three maximal apneas, respectively. During the maximal apneas, divers endured significantly more IBMs compared to preparatory apneas (28.57 ± 13.47–30.00 ± 11.32, *p* < 0.001). Details and changes in physiological parameters during the maximal apneas can be observed in Table 2.

**Figure 4:**
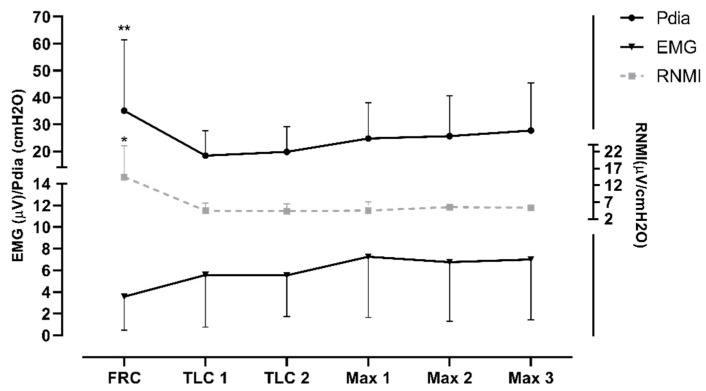
Differences between apneas in diaphragmatic pressure (Pdia), electromyographic activity (EMG), and the respiratory neuromuscular output index (RNMI) as an average of the whole struggle phase; * represents significance between FRC preparatory apnea and maximal apneas; ** represents significance with all apneas.

The diaphragm activity (i.e., EMG and Pdia) was significantly greater during the preparatory apnea at FRC compared to the preparatory apneas at TLC and the maximal apneas (*p* < 0.001; ES = 1.32–1.55). Furthermore, the RNMI was the lowest in the preparatory apnea at FRC compared to the maximal apneas (see Figure 4). The results indicate a trend of increase in EMG activity and of decrease in Pdia during the protocol.

**Figure 5:**
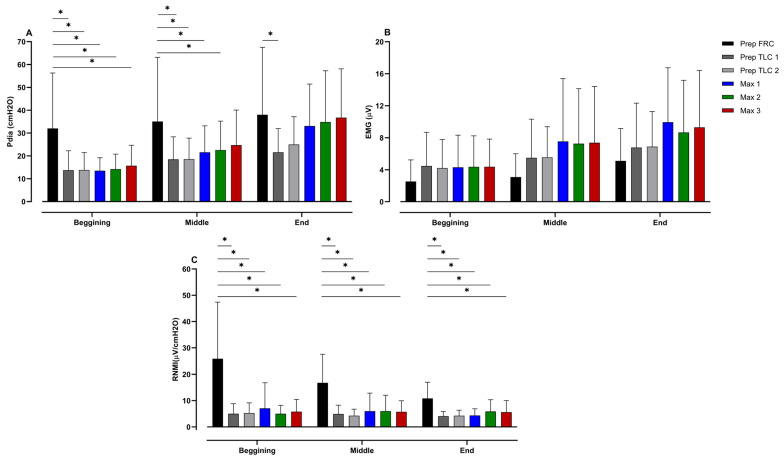
Differences between apneas in (**A**) diaphragmatic pressure (Pdia), (**B**) electromyographic activity (EMG), and (**C**) respiratory neuromuscular output index (RNMI) during the struggle phase for the beginning, middle and end periods; * represents significant differences among apneas. The apneas are shown as the functional residual capacity (FRC) and total lung capacity (TLC) for preparatory apneas and numbered in order (1–3) for both preparatory and maximal apneas.

Changes in Pdia, EMG, and the RNMI from the beginning, middle and end of the struggle phase of each apnea can be seen in Figure 5. These data show that Pdia at the onset of the struggle phase differs significantly between the preparatory apnea at FRC and the other apneas (*p* < 0.001; ES = 0.91–1.07). Likewise, Pdia in the middle of the preparatory apnea at FRC was greater than during the other preparatory apneas, as well as the first and second maximal apneas (*p* = 0.01–0.04; ES = 0.64–0.80). At the end of the struggle phase, Pdia during the preparatory apnea at FRC differed only from the first preparatory apnea at TLC (*p* = 0.04; ES = 0.76). The RNMI at the onset of the preparatory apnea at FRC was significantly lower than that for all other apneas (*p* < 0.001; ES = 1.15–1.39), and equal during the middle of the struggle phase (*p* < 0.001; ES = 1.19–1.61).

**Figure 6:**
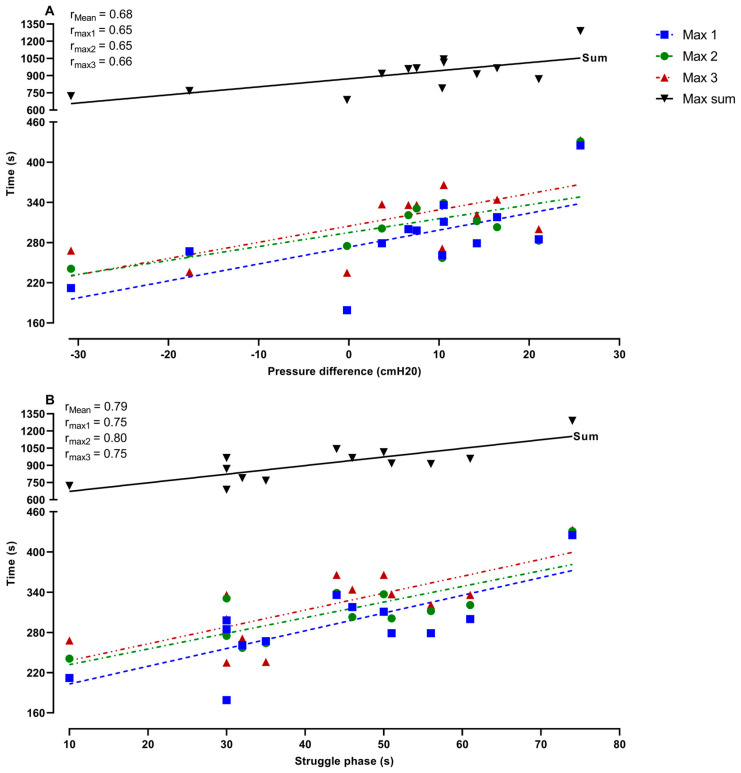
Correlations between (**A**) diaphragmatic pressure difference, and (**B**) struggle phase duration with the total duration of maximal apneas. The apneas are shown as functional residual capacity (FRC) and total lung capacity (TLC) for preparatory apneas and numbered in order (1–3) for both preparatory and maximal apneas.

Figure 6 represents the relationship between the duration of the maximal apneas and the change in Pdia (A), and the duration of the struggle phase during the preparatory apnea at FRC (B). These data demonstrate that the greater and more pronounced increase in Pdia during IBMs in the preparatory apnea at FRC was related to the longer maximal apnea times (Max 1, *p* = 0.01; Max 2, *p* = 0.02; Max 3, *p* = 0.01; Max sum, *p* = 0.01). Furthermore, the duration of the struggle phase in the preparatory apnea at FRC was also significantly related to longer maximal apnea times (Max 1, *p* ≤ 0.001; Max 2, *p* ≤ 0.001; Max 3, *p* ≤ 0.001; Max sum, *p* ≤ 0.001).

**Figure 7:**
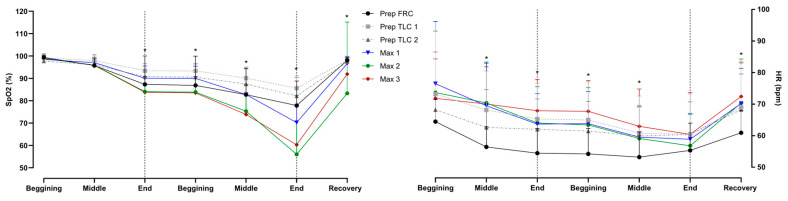
Differences between apneas in peripheral oxygen saturation (SpO_2_) and heart rate (HR), during the easy-going and struggle phase for beginning, middle and end periods; * represents significant differences. The apneas are shown as the functional residual capacity (FRC) and total lung capacity (TLC) for preparatory apneas and numbered in order (1–3) for both preparatory and maximal apneas.

The heart rate and SpO_2_ are shown in Figure 7. Divers had significantly lower SpO_2_ in maximal apneas at the end of the easy-going phase, throughout the struggle phase, and in recovery. Reductions in heart rate were present throughout the struggle phase.

## 4. Discussion

This study is the first to report the physiological changes during preparatory apneas and their association with the duration of dry, static maximal apneas. Our primary findings are as follows: (1) a greater diaphragmatic pressure difference during the struggle phase of the preparatory apnea at FRC was correlated with a longer maximal apnea duration; (2) Pdia was greater and the RNMI was the lowest during the preparatory apnea at FRC compared to the other apneas; and (3) the RNMI increased throughout the entire apnea protocol, which suggests greater nervous system activation and possible pre-activation potentiation of the diaphragm, induced by a warm-up (e.g., preparatory apnea).

Previous studies have shown that there is a short-term effect of repeated apneas on increasing the duration of a maximal apnea [19]. However, the mechanism(s) responsible for prolonging apnea duration have not been identified. According to Bain, Drvis, Dujic, MacLeod and Ainslie [2], the preparations (i.e., relaxation, diet, ventilation, metabolism, muscle tonus, HR, O_2_, and CO_2_ economy) preceding a maximal apnea are an important aspect of prolonging the duration of apnea. Importantly, these factors can be improved with apneic training, but divers can also perform acute preparations (warm-up protocols, i.e., preparatory apneas) to increase their time without breathing. Schagatay [12] stated that many elite-level BHDs perform preparatory apneas to improve their performance. Previous research suggests that preparatory apneas and relaxation techniques are used to attenuate oxygen consumption [2]. Our data shows that divers who experienced greater changes in Pdia during the preparatory apnea at FRC had longer duration maximal apneas. Additionally, many divers in our study improved their personal best maximal dry, static apnea duration. Most of the divers in this study were not competitive freedivers, and they usually do not perform preparatory apneas before their dives. Hence, the influence of preparations is highly visible in their results, since they achieved a personal best apnea time as part of the study.

Anecdotally, divers reported “softer” IBMs during the max apneas when preparatory apneas were performed. This diaphragm preparation may allow divers to endure the struggle phase of a maximal apnea with more subjective ease (authors’ correspondence with divers) and to postpone the domination of sympathetic nervous system activation. The rationale behind these findings lies in the mechanism underlying IBMs. IBMs, concomitantly with peripheral vasoconstriction-mediated centralization of the blood volume and hypercapnia-induced cerebral vasodilatation, maintain cerebral oxygenation throughout the struggle phase, thus prolonging the maximal apnea time [10]. This implies that preparatory apneas aid divers in maintaining IBMs during maximal apnea at a certain magnitude to satisfy their influence on hemodynamics, but not to overcome the ability to endure the struggle phase and prolong the breakpoint of apnea. As shown previously, the hemodynamic and respiratory functions of IBMs are well known [6]. Regarding their beneficial influence, IBMs are a potent mechanical component of the stimulus to resume breathing. Therefore, paralysis of respiratory muscles can increase tolerance to the absence of ventilation in comparison to voluntary apnea [20,21]. Accordingly, the initiation of the first IBM is termed the ‘physiological breakpoint’ [20], and is likely when naive apneists terminate the breath-hold. In contrast, elite apneists reach the break point after experiencing ~75 intensifying IBMs, well beyond the ‘physiological breakpoint’ [2]. Independent of the apneists’ ability, enduring IBMs and their intensification present a breakpoint in apnea. Therefore, the capability of divers to postpone IBMs and prolong the easy-going phase could be an explanation for apnea prolongation in our study.

A previous study performed on BHDs demonstrated that divers experience IBMs well above the previously determined diaphragm fatigue threshold [22]. In the aforementioned study, the energetic demand of muscular contractions produced by the diaphragm and inspiratory rib cage muscles rises by approximately 5- and 15-fold, respectively, from the beginning to the end of the struggle phase. Nonetheless, the divers in our study experienced increasing diaphragmatic pressure from the beginning to the end of the struggle phase. However, the results of our previous work indicated that the presence of fatigue was observed in some (~50%), but not all, divers, following our apnea protocol [14]. The lack of fatigue, shown in the previous study, could be influenced by the presence of the preparatory apneas. The already mentioned “softening” of the diaphragm can also be seen when comparing our results with the previous study. Specifically, the divers in the study by Cross et al. [22] performed two preparatory TLC apneas before performing a maximal apnea. Therefore, it can be assumed that the divers of our study benefited from the FRC preparatory apnea, which provoked the strongest Pdia for a shorter duration. Studies using MRI on diaphragm movements during the struggle phase of FRC and TLC apneas showed that IBMs at FRC do not differ from IBMs at TLC [23]. Also, the diaphragm actively participates in IBM occurrence with a similar increase in the range of diaphragmatic excursions toward the end of the struggle phase of TLC and FRC apneas [23]. The results of these studies are similar to our results, where it can be seen that Pdia is similar in the end phase of an apnea. Regardless, the preparatory apnea at FRC elicited the greatest Pdia considering the number of IBMs endured by apneists. Perhaps enduring very forceful IBMs beforehand helps apneists to endure them later on during maximal breath holds, and to prepare the respiratory neuromuscular output for the upcoming apneas.

There was an increase in the EMG signal throughout, which suggests an increase in nervous system activity for the same change in Pdia. According to Lowery [24], the recruitment of additional motor units or an increase in the firing rate of active neuronal units will cause the surface EMG amplitude signal to increase. Thus, our EMG data may be explained by the position of the diaphragm at FRC and TLC and/or the relaxation of respiratory muscles. Firstly, Batinic, Mihanovic, Breskovic, Zubin-Maslov, Lojpur, Mijacika and Dujic [23] measured diaphragm MRI during apneas at FRC and TLC. Even though both apneas showed a similar magnitude of diaphragm excursions, intrathoracic pressure was lower during apneas performed at FRC, where the diaphragm is at the optimal position on its length–tension curve [23]. Therefore, lower EMG during the preparatory apnea at FRC could imply that a smaller number of muscle fibers of the diaphragm needed to be recruited during this apnea. Secondly, Aliverti et al. [25] measured the pressures and power of the diaphragm, rib cage, and abdominal muscles during quiet breathing and exercise at 0, 30, 50, and 70% of the maximum workload. Their data suggests that the gradual relaxation of abdominal muscles during inspiration allowed the rib cage to expand and for Pdia to decrease. Following that, this might indicate a similar trend from the preparatory to maximal apneas. Precisely, the preparatory apnea at FRC may provide the necessary stimulus to stress the diaphragm the most so that BHD can endure the struggle phase more easily afterwards. Simultaneously, the nervous system output, i.e., EMG, is the lowest, which produces an increase in the activity of the nervous system for proceeding with pressure. Additionally, recent data in elite apneists reported that increased sympathetic nervous system activity is responsible for increased peripheral vasoconstriction and decreased peripheral blood flow, which presumably allows for better O_2_ economy during apneas [26,27]. Following that, the fluctuation in the respiratory muscle EMG signal from preparatory to maximal apneas indicates an increase in activity in the sympathetic nervous system, better preparation for prolonged apneas and better O_2_ economy.

### Limitations

The main limitation of this study was the lack of maximal apneas performed without the preparatory apneas. Such a protocol would be more precise in the determination of the impact of preparatory apneas on the duration of maximal apnea. Additionally, inter-individual variability in respiratory muscle strength and endurance was not observed [13]. Such results might have shown differences in muscle strength and hence influence Pdia and RNMI results, as these are dependent on contractile capacity [28]. Such measurements should be included in future studies as they might explain the relationship between preparatory and maximal apneas. Furthermore, the diving response is more pronounced during water immersion apneas or with face cooling, which was not conducted during this study. These data are part of a larger overall study [13], and thus, water immersion was not possible. Furthermore, the IBMs endured during apneas are induced by other respiratory muscles of the thorax, and not just the diaphragm [22]. Hence, obtaining EMG data on other muscles used for breathing (e.g., rectus abdominis) would be of value, since previous studies have shown different patterns of activation in the aforementioned muscles [29]. Nevertheless, the participants of this study were well-trained, with a mean apnea duration well above 5 min, so our participant sample is highly representative and indicates the strength of this study. Also, this is the first study that has explored the dynamics and possible association of preparatory apneas as an important acute factor for the prolongation of maximal apneas.

## 5. Conclusions

The present study aimed to investigate the association of preparatory apneas with the duration of maximal apneas via changes in diaphragmatic pressure and EMG data. The most important result of this study was that greater changes in Pdia during the preparatory apnea at FRC were correlated with a longer maximal apnea time. Likewise, Pdia was the greatest and the RNMI was the lowest during the preparatory apnea at FRC. Such findings indicate an acute preconditioning effect of preparatory apneas on nervous system activation and possible pre-activation potentiation of the diaphragm. Even though divers perform multiple different training strategies for maximal breath-holds (stretching, apneic training, etc.), preparatory apneas present a means to acutely increase the duration of a maximal apnea, and thus may be of value for those who do not already perform them.

## Figures and Tables

**Table 1:** Demographic data of participants (n = 14).

Variables	Mean ± SD	Range
Age (years)	39 ± 10	(22–55)
Body fat (%)	14.4 ± 3.8	(9.0–23.4)
Body height (cm)	186.6 ± 3.9	(180.0–193.3)
Body mass (kg)	87.2 ± 8.5	(69.8–101.1)
Training experience (years)	14.2 ± 9.6	(1–35)
Best static apnea (min)	5.5 ± 1.8	(3.0–10.0)
FEV1, % predicted	107.4 ± 11.1	(91–125)
FVC, % predicted	106.5 ± 11.5	(84–125)
FEV1/FVC ratio, % predicted	99.9 ± 8.4	(87–115)

SD, standard deviation; FEV1, forced expiratory volume in 1 s; FVC, forced vital capacity.

**Table 2:** Differences between preparatory and maximal apneas in the duration of apneas and the time tension index.

Variables	Prep FRC	Prep TLC 1	Prep TLC 2
	Mean ± SD	Mean ± SD	Mean ± SD
Total Duration (s)	117.57 ± 41.77	213.00 ± 53.36 *^,××^	223.50 ± 55.40 *^,××^
Easy-going phase (s)	77.71 ± 38.33	153.86 ± 37.34 *^,××^	164.07 ± 41.94 *^,××^
Struggle phase (s)	39.86 ± 18.35	59.14 ± 27.40 ^××^	59.43 ± 30.51 ^××^
Number of IBMs	8.23 ± 2.83	10.50 ± 6.87	12.57 ± 9.72
Pdia (cmH_2_O)	35.11 ± 26.29	18.42 ± 9.24 *^,×^	19.81 ± 9.30 *^,×^
EMG (µV)	3.57 ± 3.07	5.58 ± 4.82	5.55 ± 3.81
RNMI (μV/cmH_2_O)	14.48 ± 9.16	4.48 ± 2.27 *	4.37 ± 2.13 *^,××^
	Max 1	Max 2	Max 3
Total Duration (s)	291.74 ± 58.07 *^,¥,××^	310.98 ± 48.71 *^,¥,××^	322.15 ± 55.33 *^,¥,××^
Easy-going phase (s)	180.36 ± 44.84 *^,¥,××^	202.36 ± 50.80 *^,¥,××^	201.60 ± 50.06 *^,¥,××^
Struggle phase (s)	111.38 ± 33.48 ^¥,××^	108.62 ± 31.65 *^,¥,××^	120.54 ± 34.70 *^,¥,××^
Number of IBMs	28.57 ± 13.47 ^¥,××^	30.00 ± 11.32 *^,¥,××^	28.71 ± 12.58 *^,¥,××^
Pdia (cmH_2_O)	24.75 ± 13.28	25.61 ± 15.04	27.73 ± 17.65
EMG (µV)	7.26 ± 5.63	6.76 ± 5.47	7.01 ± 5.57
RNMI (μV/cmH_2_O)	4.55 ± 2.55 *^,××^	5.54 ± 3.81 *^,××^	5.38 ± 3.72 *^,××^

Prep, preparatory; Max, maximal; FRC, functional residual capacity; TLC, total lung capacity; RNMI, respiratory neuromuscular output index; IBM, involuntary breathing movements; SD, standard deviation; *, significant difference with preparatory apnea FRC; ^¥^, significant difference with preparatory apneas TLC; ^×^, moderate effect size; ^××^, large effect size.

## Data Availability

The original contributions presented in this study are included in the article. Further inquiries can be directed to the corresponding author.

## Abbreviations

The following abbreviations are used in this manuscript:

BHD	Breath hold divers
EMG	Electromyography
IBM	Involuntary breathing movements
Pdi	Diaphragm pressure
RNMI	Respiratory neuromuscular output index
FRC	Functional residual capacity
DR	Diving response
MAP	Mean arterial pressure
HR	Heart rate
